# Liver cancer risk, coffee, and hepatitis C virus infection: a nested case–control study in Japan

**DOI:** 10.1038/sj.bjc.6603891

**Published:** 2007-07-17

**Authors:** K Wakai, Y Kurozawa, A Shibata, Y Fujita, K Kotani, I Ogimoto, M Naito, K Nishio, H Suzuki, T Yoshimura, A Tamakoshi

**Affiliations:** 1 Department of Preventive Medicine/Biostatistics and Medical Decision Making, Nagoya University Graduate School of Medicine, 65 Tsurumai-cho, Showa-ku, Nagoya 466-8550, Japan; 2 Department of Social Medicine, Division of Health Administration and Promotion, Faculty of Medicine, Tottori University, Nishimachi 86, Yonago 683-8503, Japan; 3 Department of Public Health, Kurume University School of Medicine, 67 Asahi-machi, Kurume 830-0011, Japan; 4 Department of Public Health, Graduate School of Medical and Dental Sciences, Niigata University, 1-757 Asahimachi-dori, Niigata 951-8510, Japan; 5 Fukuoka Institute of Health and Environmental Sciences, 39 Oaza-Mukaizano, Dazaifu 818-0135, Japan

**Keywords:** coffee, hepatocellular carcinoma, hepatitis C virus, cohort study, nested case–control study

## Abstract

We examined hepatocellular carcinoma mortality in relation to coffee consumption and anti-hepatitis C virus (HCV) antibody seropositivity in a nested case–control study involving 96 cases. The multivariate-adjusted odds ratios (95% confidence interval) for daily coffee drinkers *vs* non-drinkers were 0.49 (0.25–0.96), 0.31 (0.11–0.85), and 0.75 (0.29–1.92) in all cases, in HCV-positive and in HCV-negative individuals, respectively.

The inverse associations between coffee consumption and the risk of hepatocellular carcinoma (HCC) have recently been reported not only from case–control studies (Gallus *et al*, 2002; Gelatti *et al*, 2005; Ohfuji *et al*, 2006; Montella *et al*, 2007; Tanaka *et al*, 2007) but also from Japanese cohort studies (Inoue *et al*, 2005; Kurozawa *et al*, 2005; Shimazu *et al*, 2005). Cohort studies are superior to case–control studies in avoiding recall and selection bias (Ohfuji *et al*, 2006). Previous prospective studies (Inoue *et al*, 2005; Kurozawa *et al*, 2005; Shimazu *et al*, 2005), however, did not consider the infection status of hepatitis C virus (HCV) at baseline. As HCV is the major cause of HCC in Japan and certain other countries (Heathcote, 2004), it would be important if protective factors against HCC could be found among the HCV-positive population. We therefore examined the relation of coffee use to risk of death from HCC by HCV infection status in a case–control study nested in a large cohort study in Japan.

## MATERIALS AND METHODS

We carried out a nested case–control study as a part of the Japan Collaborative Cohort Study for Evaluation of Cancer Risk Sponsored by the Ministry of Education, Culture, Sports, Science and Technology of Japan (Mobusho), details of which are described elsewhere (Tamakoshi *et al*, 2005). It involved 110 792 individuals, aged 40–79 years at baseline, from 45 areas throughout Japan. A self-administered questionnaire on lifestyle and medical factors was distributed in 1988–1990 covering habitual coffee consumption, with possible responses including ‘scarcely any’, ‘1–2 cups per month’, ‘1–2 cups per week’, ‘3–4 cups per week’, and ‘almost every day’. Those who answered ‘almost every day’ were asked to report the number of cups consumed per day. The questionnaire was validated using four 3-day dietary records as a reference; the Spearman correlation coefficient was 0.79 (Iso *et al*, 2006).

In addition, those participants who underwent health-screening checks sponsored by municipalities were asked to donate blood samples at baseline and eventually, 39 242 subjects in 37 study areas did so (Tamakoshi *et al*, 2005), these being stored at −80°C until analysed. Informed consent was obtained individually from subjects, except in certain areas in which it was provided at the group level after details had been explained to community leaders. The Ethics Committee of Kurume University School of Medicine approved this study.

We used population registries in the municipalities to determine the vital and residential status of the subjects. Causes of death were confirmed by review of death certificates with permission from the Ministry of Internal Affairs and Communications. Cases eligible for the present study consisted of those who died of HCC, ICD-10 coded C22.0.

During follow-up through the end of 1999, 106 eligible cases were identified among the participants with serum samples, from which two were excluded with insufficient samples and eight without information on coffee consumption. Of the remaining 96 cases, 60 (62.5%) were positive for HCV Ab. As potential controls, sera of 11 513 subjects from the same geographical areas as the cases were also screened for HCV Ab. After excluding those with missing data on coffee drinking, we found 912 HCV-Ab-positive subjects (8.2%) and 10 175 HCV-Ab-negative ones. From these, we chose as many controls per case as possible, matching for age (same 5-year strata), sex, and HCV-Ab seropositivity, selecting 420 HCV-Ab-positive controls (seven controls per case) and 3024 HCV-Ab-negative ones (84 controls per case).

### Statistical analysis

Study participants were categorised into three groups by coffee consumption, that is, ⩾1 cup day^−1^, <1 cup day^−1^ (‘1–2 cups month^−1^’, ‘1–2 cups week^−1^’, or ‘3–4 cups week^−1^’), and non-drinkers. Daily drinkers could not be further subdivided because of their small numbers. Odds ratios (OR) and the 95% confidence intervals (CI) by HCV-Ab positivity were estimated considering the matching using conditional logistic models (Breslow and Day, 1980). Multivariate-adjusted OR were also computed after adjustment for area, smoking and drinking habits, and history of diabetes mellitus and liver diseases. For alcohol drinking, subjects were categorised into never drinkers, former drinkers, or current drinkers who consumed <2 or ⩾2 Japanese drinks per day (one Japanese drink is equivalent to 23 g of ethanol) in this analysis. The linear trend in HCC risk was tested by treating the coffee consumption category as an ordinal variable. The heterogeneity in the association of coffee drinking by HCV status was statistically tested by incorporating a multiplicative interaction term between HCV status and the coffee consumption category in the model. Missing values for each covariate were treated as an additional category in the variable and were included in the model. All *P*-values were two-sided, and all the analyses were carried out using the Statistical Analysis System version 9.1 (SAS Institute, Cary, NC, USA).

## RESULTS

Cases and controls were well matched on age and sex in both the HCV-positive and -negative groups. The mean ages±s.d. were 62.9±6.6, 62.4±6.2, 63.6±7.5, and 63.4±7.3 years in the HCV-positive cases and controls and the HCV-negative cases and controls, respectively. Women accounted for 35.0% of both the HCV-positive cases and controls, and 36.1% of the HCV-negative cases and controls. Case subjects were more likely to currently smoke than controls in the HCV-positive group (56.6 *vs* 35.8%). Former drinkers and a history of diabetes mellitus and liver diseases were much more common in cases than in controls. In the HCV-positive cases, the proportions of former drinkers and those with diabetes and liver diseases were 28.6, 15.0, and 56.7%, respectively, against 6.6, 5.0, and 20.7% in the controls. The corresponding figures were 14.3, 13.9, and 27.8% in the HCV-negative cases and 4.4, 4.5, and 4.4% in the controls.

Drinking one or more cups of coffee per day was inversely associated with HCC mortality among all subjects (Table 1: multivariate-adjusted OR (OR2), 0.49; 95% CI, 0.25–0.96) and the anti-HCV-positive group (OR2, 0.31; 95% CI, 0.11–0.85). Although daily coffee drinkers in the HCV-negative group showed OR below unity, they did not reach statistical significance. The heterogeneity in the association of coffee drinking by HCV status was also not significant in the multivariate model (*P* = 0.61).

## DISCUSSION

Coffee drinking was significantly associated with a decreased risk of death from HCC in all subjects and those infected with HCV. Our results from this prospective cohort study support the findings in some (Gelatti *et al*, 2005; Ohfuji *et al*, 2006), although not all (Montella *et al*, 2007), case–control studies that suggested a protective effect of coffee among HCV-positive individuals. Some patients with hepatitis or liver cirrhosis, however, may have decreased coffee consumption at their physician's advice or due to impaired caffeine metabolism in the liver (Hasegawa *et al*, 1989). Observational studies among subjects without active hepatitis or intervention studies will further clarify the role of coffee in the possible prevention of HCV-related HCC. Further, because a nonsignificant inverse association was found between coffee consumption and HCC risk in HCV-negative individuals in the present study, investigations with more HCV-negative HCC cases are also warranted.

## Figures and Tables

**Table 1:** OR and 95% CI for HCC mortality according to coffee consumption by anti-HCV-antibody seropositivity

	**All subjects**						**Anti-HCV-positive group**						**Anti-HCV-negative group**					
**Coffee consumption**	**No. of cases**	**No. of controls**	**OR1^a^**	**95%CI**	**OR2^b^**	**95%CI**	**No. of cases**	**No. of controls**	**OR1^c^**	**95%CI**	**OR2^b^**	**95%CI**	**No. of cases**	**No. of controls**	**OR1^c^**	**95%CI**	**OR2^b^**	**95%CI**
Non-drinkers	44	1163	1.00		1.00		28	147	1.00		1.00		16	1016	1.00		1.00	
<1 cup day^−1^	34	1266	0.72	0.45–1.15	0.77	0.45–1.32	23	153	0.79	0.44–1.42	0.91	0.41–2.04	11	1113	0.62	0.29–1.35	0.65	0.29–1.46
⩾1 cup day^−1^	18	1015	0.49	0.28–0.86	0.49	0.25–0.96	9	120	0.39	0.18–0.87	0.31	0.11–0.85	9	895	0.63	0.28–1.45	0.75	0.29–1.92
*P*-value for trend				0.012		0.038				0.022		0.031				0.24		0.45

CI, confidence intervals; OR, odds ratios.

a Considered for age, sex, and anti-HCV-antibody seropositivity using a conditional logistic model.

b Further adjusted for area, smoking and drinking habits, and history of diabetes mellitus and liver diseases.

c Considered for age and sex using a conditional logistic model.

## Appendix A

*Japan Collaborative Cohort Study Group* The present investigators involved, with the co-authorship of this paper, in the JACC Study and their affiliations are as follows: Dr Akiko Tamakoshi (present chairman of the study group), Nagoya University Graduate School of Medicine; Dr Mitsuru Mori, Sapporo Medical University School of Medicine; Dr Yutaka Motohashi, Akita University School of Medicine; Dr Ichiro Tsuji, Tohoku University Graduate School of Medicine; Dr Yosikazu Nakamura, Jichi Medical School; Dr Hiroyasu Iso, Graduate School of Medicine, Osaka University; Dr Haruo Mikami, Chiba Cancer Center; Dr Yutaka Inaba, Juntendo University School of Medicine; Dr Yoshiharu Hoshiyama, University of Human Arts and Sciences; Dr Hiroshi Suzuki, Niigata University School of Medicine; Dr Hiroyuki Shimizu, Gifu University School of Medicine; Dr Hideaki Toyoshima and Dr Kenji Wakai, Nagoya University Graduate School of Medicine; Dr Shinkan Tokudome, Nagoya City University Graduate School of Medical Sciences; Dr Yoshinori Ito, Fujita Health University School of Health Sciences; Dr Shuji Hashimoto, Fujita Health University School of Medicine; Dr Shogo Kikuchi, Aichi Medical University School of Medicine; Dr Akio Koizumi, Graduate School of Medicine and Faculty of Medicine, Kyoto University; Dr Takashi Kawamura, Kyoto University Center for Student Health; Dr Yoshiyuki Watanabe, Kyoto Prefectural University of Medicine, Graduate School of Medical Science; Dr Tsuneharu Miki, Graduate School of Medical Science, Kyoto Prefectural University of Medicine; Dr Chigusa Date, Faculty of Human Life and Environment, Nara Women's University; Dr Kiyomi Sakata, Wakayama Medical University; Dr Youichi Kurozawa, Tottori University Faculty of Medicine; Dr Norihiko Hayakawa, Research Institute for Radiation Biology and Medicine, Hiroshima University; Dr Takesumi Yoshimura, Fukuoka Institute of Health and Environmental Sciences; Dr Akira Shibata, Kurume University School of Medicine; Dr Naoyuki Okamoto, Kanagawa Cancer Center; Dr Hideo Shio, Moriyama Municipal Hospital; Dr Yoshiyuki Ohno, Asahi Rosai Hospital; Dr Tomoyuki Kitagawa, Cancer Institute of the Japanese Foundation for Cancer Research; Dr Toshio Kuroki, Gifu University; and Dr Kazuo Tajima, Aichi Cancer Center Research Institute.

## References

[bib1] Breslow NE, Day NE (1980) Conditional logistic regression for matched sets. In Davis W. (ed) Statistical Methods in Cancer Research vol 1. International Agency for Research on Cancer: Lyon, pp 248–279

[bib2] Gallus S, Bertuzzi M, Tavani A, Bosetti C, Negri E, La Vecchia C, Lagiou P, Trichopoulos D (2002) Does coffee protect against hepatocellular carcinoma? Br J Cancer 87: 956–959, doi: 10.1038/sj.bjc.6600582 12434283 10.1038/sj.bjc.6600582 PMC2364316

[bib3] Gelatti U, Covolo L, Franceschini M, Pirali F, Tagger A, Ribero ML, Trevisi P, Martelli C, Nardi G, Donato F (2005) Coffee consumption reduces the risk of hepatocellular carcinoma independently of its aetiology: a case–control study. J Hepatol 42: 528–534, doi: 10.1016/j.jhep.2004.11.039 15868652 10.1016/j.jhep.2004.11.039

[bib4] Hasegawa M, Yamada S, Hirayama C (1989) Fasting plasma caffeine level in cirrhotic patients: relation to plasma levels of catecholamines and renin activity. Hepatology 10: 973–977 2684839 10.1002/hep.1840100614

[bib5] Heathcote EJ (2004) Prevention of hepatitis C virus-related hepatocellular carcinoma. Gastroenterology 127: S294–S302, doi: 10.1053/j.gastro.2004.09.044 15508097 10.1053/j.gastro.2004.09.044

[bib6] Inoue M, Yoshimi I, Sobue T, Tsugane S (2005) Influence of coffee drinking on subsequent risk of hepatocellular carcinoma: a prospective study in Japan. J Natl Cancer Inst 97: 293–300, doi: 10.1093/jnci/dji040 15713964 10.1093/jnci/dji040

[bib7] Iso H, Date C, Wakai K, Fukui M, Tamakoshi A (2006) The relationship between green tea and total caffeine intake and risk for self-reported type 2 diabetes among Japanese adults. Ann Intern Med 144: 554–562 16618952 10.7326/0003-4819-144-8-200604180-00005

[bib8] Kurozawa Y, Ogimoto I, Shibata A, Nose T, Yoshimura T, Suzuki H, Sakata R, Fujita Y, Ichikawa S, Iwai N, Tamakoshi A (2005) Coffee and risk of death from hepatocellular carcinoma in a large cohort study in Japan. Br J Cancer 93: 607–610, doi: 10.1038/sj.bjc.6602737 16091758 10.1038/sj.bjc.6602737 PMC2361599

[bib9] Montella M, Polesel J, La Vecchia C, Dal Maso L, Crispo A, Crovatto M, Casarin P, Izzo F, Tommasi LG, Talamini R, Franceschi S (2007) Coffee and tea consumption and risk of hepatocellular carcinoma in Italy. Int J Cancer 120: 1555–1559, doi: 10.1002/ijc.22509 17205531 10.1002/ijc.22509

[bib10] Ohfuji S, Fukushima W, Tanaka T, Habu D, Tamori A, Sakaguchi H, Takeda T, Kawada N, Seki S, Nishiguchi S, Shiomi S, Hirota Y (2006) Coffee consumption and reduced risk of hepatocellular carcinoma among patients with chronic type C liver disease: a case–control study. Hepatol Res 36: 201–208, doi: 10.1016/j.hepres.2006.07.010 16916617 10.1016/j.hepres.2006.07.010

[bib11] Shimazu T, Tsubono Y, Kuriyama S, Ohmori K, Koizumi Y, Nishino Y, Shibuya D, Tsuji I (2005) Coffee consumption and the risk of primary liver cancer: pooled analysis of two prospective studies in Japan. Int J Cancer 116: 150–154, doi: 10.1002/ijc.20989 15756689 10.1002/ijc.20989

[bib12] Tamakoshi A, Yoshimura T, Inaba Y, Ito Y, Watanabe Y, Fukuda K, Iso H (2005) Profile of the JACC study. J Epidemiol 15(Suppl 1): S4–S8 15881191 10.2188/jea.15.S4 PMC8565873

[bib13] Tanaka K, Hara M, Sakamoto T, Higaki Y, Mizuta T, Eguchi Y, Yasutake T, Ozaki I, Yamamoto K, Onohara S, Kawazoe S, Shigematsu H, Koizumi S (2007) Inverse association between coffee drinking and the risk of hepatocellular carcinoma: a case-control study in Japan. Cancer Sci 98: 214–218, doi: 10.1111/j.1349-7006.2006.00368.x 17233838 10.1111/j.1349-7006.2006.00368.x PMC11159716

